# Race and socioeconomic effect on sarcopenia and sarcopenic obesity in the Louisiana Osteoporosis Study (LOS)

**Published:** 2018

**Authors:** Cassie Jeng, Lan-Juan Zhao, Kehao Wu, Yu Zhou, Ted Chen, Hong-Wen Deng

**Affiliations:** 1 Western Michigan University, 1903 W. Michigan Ave., Kalamazoo, MI 49008-5378; 2 Tulane University, 1440 Canal St., Suite 2001, New Orleans, LA 70112

**Keywords:** Prevalence rates, Race and ethnicity, Sarcopenia, Sarcopenic obesity, Socioeconomic factors

## Abstract

**Background:**

Sarcopenia and sarcopenic obesity are emerging public health issues. True prevalence rates are unknown and estimates differ substantially between studies. No large-scale single study has compared prevalence rates between whites, blacks, Asians, and Hispanics, as we intend to do here. This study also examined the effects of race and socioeconomic factors on sarcopenia and sarcopenic obesity.

**Methods:**

This study included 10,325 participants from Louisiana. Appendicular lean mass (ASM), measured through dual energy x-ray absorptiometry (DXA) scans, was divided by height squared (ASM/h^2^) to define sarcopenia. Sarcopenic obesity was defined as sarcopenia plus obesity (waist-to-hip ratio).

**Results:**

Overall sarcopenia and sarcopenic obesity rates were 17.6% and 7.0% for males, and 13.7% and 2.5% for females, respectively. The highest sarcopenia and sarcopenic obesity rates were found in Asian males (40.6%, 14.4%) and females (30.1%, 8.0%). The lowest sarcopenic obesity rates were observed in black males (3.7%) and females (0.9%). We found significant associations with sarcopenic obesity in males for age, race, and income; in females, for age, race, and education.

**Conclusions:**

Under one diagnostic definition, the prevalence of sarcopenia and sarcopenic obesity is highest among Asians and lowest amongst blacks. Income and education had significant associations with sarcopenia and sarcopenic obesity, in males and females, respectively.

## Introduction

As the first of the “baby boomer” generation started turning 65 in 2011 [1], the elderly population in the United States is expected to increase from 35 million in 2000 [2], to 74 million (20.6% of the population) in 2030 [3]. Sarcopenia – generally defined as the age-related decrease in skeletal muscle mass [4–5] – has been found to be associated with many adverse health outcomes, including metabolic issues [6–8] and physical disability [4, 9]. Sarcopenic obesity – generally defined as the co-occurrence of sarcopenia and obesity [10–11] – has a combined effect that increases the severity of both health conditions [12–13], leading to worse functional declines, more physical disabilities, and poorer health outcomes [14–17].

The true prevalence rates of sarcopenia and sarcopenic obesity are unknown. Due to the use of different definitions, reported prevalence rates for sarcopenia and sarcopenic obesity differ substantially among studies [12, 18]. While six major “consensus” definitions have been published by various international special interest and working groups, there is no real consensus for a diagnostic definition of sarcopenia and sarcopenic obesity [19–24].

Race and ethnicity may explain some of the high variation of prevalence rates for sarcopenia and sarcopenic obesity. It is well-established that body composition differs between major races [25–28], and the discussion about race-specific cutoffs is building. For example, the World Health Organization (WHO) has proposed lowering body mass index (BMI) cutoffs for Asian populations, from normal standards of overweight: 25–29.9 kg/m^2^ and obese: ⩾30 kg/m^2^ to adjusted standards of overweight: 23–27.4 kg/m^2^ and obese: ⩾27.5 kg/m^2^ [28–29].

In our literature review, we did not find any publication that compared the prevalence of sarcopenic obesity between whites, blacks, Asians, and Hispanics from one large-scale study using a representative young reference population. Additionally, other studies have shown that socioeconomic factors may also play a role in sarcopenic and sarcopenic obesity, but no associations are reliably found [30–33]. The aim of this study is to examine the race/ethnicity and socioeconomic effects on the prevalence rates of sarcopenia and sarcopenic obesity. Better understanding of these effects on sarcopenia and sarcopenic obesity will contribute to the discussions on specific cutoffs for different ethnicities, provide guidelines for measuring true prevalence rates, and help promote better prevention and management of sarcopenia and sarcopenic obesity.

## Methods

### Study participants

This study used collected data from the Louisiana Osteoporosis Study (LOS), which has been ongoing since 2011 [34]. The study was approved by the IRB of Tulane University and all participants signed an informed consent form.

LOS had recruited 10,475 random human subjects at the preparation of this paper. We excluded 150 participants from the data analysis due to missing anthropometric or incomplete dual energy x-ray absorptiometry (DXA) scan results. The final dataset contained 10,325 subjects, who had complete race/ethnicity, anthropometric data information, and valid muscle mass data.

### Measurements

Research staff measured height, weight, waist circumference, and hip circumference for each LOS participant. Height and weight were measured using a Health-O-Meter Professional height and weight scale, in centimeters and kilograms, respectively. Waist and hip circumferences were measured at maximal circumferences in centimeters. Whole body composition, including lean mass in both arms and legs, was measured using a DXA machine (Hologic Discovery A system, Hologic Inc., Bedford, MA, USA). Subjects self-reported as African-American/Black, Asian, Caucasian/White, and Hispanic/Latino. Participants who didn’t fit one of these categories were designated “other” for ethnicity. Personal annual income and education were optionally self-reported with categorized selections in the LOS medical questionnaire.

### Definitions

We divided the 10,325 subjects into two groups according to their ages at the time of the DXA scan, calculated from reported date of birth and scan date. Those under the age of 40 constituted the young reference group, totaling 2,663 participants (1,024 males, 1,639 females). The analyzed sample, at least 40 years old, has a total of 7,662 subjects (2,971 males, 4,691 females).

In most published studies [35], sarcopenia was defined using height-adjusted appendicular skeletal muscle mass (ASM/h^2^), with ASM calculated as the sum of the lean mass of the arms and legs. In order to compare the prevalence of sarcopenia for different racial/ethnic groups, we calculated the means of ASM/h^2^ in males and females for the entire young reference population. We defined sarcopenia as one standard deviation below the gender-specific means (males: <7.8 kg/m^2^, females: <5.88 kg/m^2^).

For this study, we selected both body mass index (BMI) and waist-to-hip ratio (WHR) to categorize obesity in order to examine how obesity rates differ by index. BMI was calculated as weight in kilograms divided by the square of height in meters. BMI cutoff values are: <25 = normal, 25–29.9 = overweight, and ⩾30 = obese [36]. WHR was calculated as ratio of the circumference of the waist to that of the hips. The WHR cutoff values for defining central obesity are: ⩾0.90 for males and ⩾ 0.85 for females [37].

Sarcopenic obesity was defined as sarcopenia plus obesity as defined by WHR. We chose WHR over BMI to reduce the possibility of overall weight misrepresenting fat mass, and to sustain the use of gender-specific thresholds, similar to sarcopenia.

### Statistical Analyses

All analyses were conducted in SAS versions 9.3 and 9.4.

Data were analyzed in sex-specific sets. Univariate analyses were conducted to calculate demographic and prevalence data across five race/ethnicity groups. Income and education were the only two optional data points, with rates of missing data ranging from 17.1% to 39.1%.

Analysis of variance (ANOVA) and group *t* tests were used to determine group differences in the anthropometric data. Multinomial stepwise regression, a variation of forward selection regression, was used to test associations between socioeconomic factors and sarcopenic obesity, as well as to calculate individual odds ratios in the analyzed population. For this last set of analyses, race/ethnicity had to be condensed into three groups due to insufficient distribution of participants (combining “Hispanic”, “Asian”, and “Other” into one “Other” group) in testing the associations of social determinant factors with sarcopenia, obesity, and sarcopenic obesity by gender. Statistical significance was defined as p-value < .05.

## Results

### Demographics

Race, income, and education levels of the 10,325 participants can be found in Table 1. The majority of our LOS subjects were white (54.3%) and black (32.2%) in both the study population (56.1% white; 32.4% black) and the young reference population (48.9% white; 31.5% black). In the female group, there were more white people in the study group (64.3%) compared to the young reference group (50%). In the male group, there were more black subjects in the study population (45.5%) compared to that in the young reference group (35.6%). The distribution of the subjects was similar for other races in the study and young reference populations.

### Anthropometrics

Anthropometric data of the study population (n=7,662) can be found in Table 2. All data were grouped by gender and examined in separate race/ethnicity groups. Each measure, which included age, BMI, WHR, and ASM/h^2^, had significant group differences across the five race/ethnicity groups and the differences are listed in the group differences column.

### Prevalence rates for obesity, sarcopenia, and sarcopenic obesity

Prevalence rates for obesity, sarcopenia, and sarcopenic obesity in subject study population can be found in Table 3.

Considering both BMI and WHR results, the highest obesity rates by race were Hispanic males (35.4% by BMI, 69.2% by WHR) and black females (54.6% by BMI, 55.4% by WHR). The lowest obesity rates by race/ethnicity using BMI were Asian males (4.8%) and Asian females (6.9%).

The overall sarcopenia rates were 17.6% for males and 13.7% for females. The highest sarcopenia rates by race/ethnicity were Asian for both males (40.6%) and females (30.1%). The lowest sarcopenia rates by race/ethnicity were Hispanic males (10.8%) and black females (3.6%).

Overall sarcopenic obesity rates were 7.0% in males and 2.5% in females. The highest sarcopenic obesity rates by race/ethnicity were Asian males (14.4%) and Asian females (8.0%). The lowest sarcopenic obesity rates by race/ethnicity were black males (3.7%) and black females (0.9%).

### Associations of socioeconomic factors with obesity, sarcopenia, and sarcopenic obesity

We tested the effects of age, race, personal annual income, and education on sarcopenic obesity, sarcopenia only, and obesity only. All examined factors, with the exception of education level in males, were found to be significant (at least p < .05) for at least one outcome (sarcopenia only, obesity only, or sarcopenic obesity). The odds ratios are presented in Table 4.

For males, age had the largest associations with sarcopenic obesity, with odds ratios increasing with age, from 3.54 (p < .001) in the 50s to as high as 18.33 (p < .001) for 70+ years of age, as compared to the 40s. Significant associations for race/ethnicity were found for blacks (as compared to white) with an odds ratio of 0.21 (p < .001) for sarcopenic obesity, 0.38 (p < .001) for sarcopenia only, and 0.46 (p < .001) for obesity only. Income was found to be significant only for sarcopenic obesity – males with a personal annual income less than $20,000 were 2.78 times more likely (p < .01) to have sarcopenic obesity than males with an annual income of $60,000 or more.

For females, age also had the largest associations with sarcopenic obesity, with odds ratios increasing from 2.26 (p < .05) in the 50s to 4.96 (p < .001) for 70+ years of age, as compared to the 40s. Significant associations for race/ethnicity were found for blacks (as compared to whites) with an odds ratio of 0.19 (p < .001) for sarcopenia only and 1.60 (p < .001) for obesity only. Looking at education levels, females with a high school degree and/or some college were 2.68 times more likely (p < .01) to have sarcopenic obesity compared to females with at least a college degree.

## Discussion

One of the key findings of this study is the prevalence rates of sarcopenia and sarcopenic obesity across the different races/ethnicities. Asians had the highest rates of both sarcopenia and sarcopenic obesity, and almost all of the lowest rates of sarcopenia and sarcopenic obesity were found in blacks. Personal annual income and education level had significant associations with sarcopenia and/or sarcopenic obesity.

The overall sarcopenic obesity prevalence rates are the lowest in both black men and black women in our study. This finding is consistent with previous findings from Batsis et al. [12]. Interestingly, our finding that Asians have the highest rates of sarcopenia and sarcopenic obesity is opposite to other studies, particularly those done in Asian countries, where the use of an Asian reference population showed very low rates of sarcopenia and sarcopenic obesity [22, 31, 38]. Because there is no consensus on a definition for sarcopenic obesity and different studies use different reference groups, it is difficult to compare the prevalence rates found here to other studies. Additionally, most studies in the United States have not had enough diversity in their study populations to examine prevalence rates beyond whites and blacks. Studies from Asian countries have used Asian young reference populations to define sarcopenia and sarcopenic obesity, making those results difficult to compare to most other non-Asian studies, which tend to have more diverse young reference populations. However, studies outside of Asia typically include very few, if any, Asians, meaning that no conclusions about Asian populations can be made in comparison to the other included races/ethnicities. Our finding that Asians had the highest prevalence rates of sarcopenia and sarcopenic obesity is significant and unique in that it is the first study to identify a local Asian population as having the highest prevalence rates of sarcopenia and sarcopenic obesity compared to local whites, blacks, and Hispanics, all under the same diagnostic criteria and definitions. Further studies should be done with more diverse populations to better understand the effect of race and ethnicity on sarcopenic obesity.

Age is an established factor of sarcopenia [4, 7, 11] and, to some extent, sarcopenic obesity [10, 11, 35]. It proved to have the strongest positive associations in our study. However, the strength of association is difficult to compare across studies because there is no consensus about how to define age groups. In our study, age had the strongest effect on sarcopenic obesity, compared to sarcopenia alone and obesity alone. The socioeconomic effects of income and education level that are found in this study are similar to findings from other studies. Alexandre et al. found that low income was a risk factor for sarcopenia [33]. In our study, low income was associated with sarcopenic obesity only in males, and sarcopenia only and obesity only in females. Hwang et al. found that having a middle school education or lower was associated with sarcopenic obesity in a Korean male population [31]. In our study, lower educational level was associated with sarcopenic obesity only in the female group. Clearly, much more research is needed in this area.

There were some design issues in our study regarding the definition of sarcopenic obesity. First, we selected WHR to define obesity in our study for two reasons: one, it provided gender-specific cutoffs; and two, it was the least contested across different races/ethnicities. However, there is no clear consensus for which index of obesity is the most useful, so any chosen index may not necessarily reflect the true prevalence of obesity. Second, we chose to define sarcopenia as <1SD below the young reference population mean, based on Janssen’s definition of class I sarcopenia [9]. Most studies use <2SD below the young reference population mean, which is class II sarcopenia by Janssen’s definition [12]. We selected <1SD for two reasons: one, the reference population data was too clustered that there were no participants classified as sarcopenic using the <2SD definition; and two, there are studies validating the use of <1SD, particularly as a way to diagnose less severe sarcopenia [39–41]. Additionally, we did not have enough data variety to use any multi-pronged definitions of sarcopenia, like the FNIH definition [23].

This study had several strengths, including: the large sample size used, the diversity of races/ethnicities examined, and the use of a local, combined, young reference population to calculate the cutoff points for defining sarcopenia. There were also several limitations to this study, including the expected high percentages of missing data for personal annual income and education level. Despite the large sample size, the distribution of outcomes across race/ethnicity groups was insufficient for complete association analysis across all five categories. As the LOS progresses, it is hoped that more data will allow for more diverse race/ethnicity-specific analyses. Second, the subjects for LOS are recruited in New Orleans and Baton Rouge and surrounding areas. Conclusions from this study may not be able to be generalized in other populations. Finally, it is hoped that future differentiation between class I and class II sarcopenia can be applied in future research in order to examine associations on multiple outcome levels.

In conclusion, under the same diagnostic criteria and definitions, our study shows that the prevalence of sarcopenia and sarcopenic obesity is highest amongst Asian men and women. Black men and women have the lowest prevalence rates of sarcopenic obesity. More studies with diverse populations need to be done to replicate the finding. Additionally, both income and education level had significant associations with sarcopenia and/or sarcopenic obesity. This study reflects the need for current research to more specifically identify, define, and measure sarcopenia and sarcopenic obesity. The age and race/ethnicity associations found in this study show that the definition of sarcopenic obesity could benefit from being more specifically defined by subgroups of populations to provide true sarcopenia and sarcopenic obesity rates.

## Figures and Tables

**Table 1:** Demographic characteristics of study and reference populations by gender.

	Male		Female	
	
	Study Population n = 2971	Young Reference n = 1024	Study Population n = 4691	Young Reference n = 1639
Race				
*White*	43.2%	47.1%	64.3%	50.0%
*Black*	45.5%	35.6%	24.2%	29.0%
*Asian*	6.3%	10.5%	7.2%	12.9%
*Hispanic*	2.2%	3.0%	2.7%	4.3%
*Other*	2.9%	3.9%	1.6%	3.8%
Salary				
*Less than $20,000*	38.0%	47.0%	17.5%	34.5%
*$20,000–$59,999*	12.8%	14.3%	26.2%	27.8%
*$60,000 or more*	10.1%	3.6%	22.6%	8.7%
*Missing*	39.1%	35.2%	33.8%	29.0%
Education				
*< H.S. degree*	13.9%	13.6%	7.8%	7.2%
*H.S. degree – some college*	38.5%	37.0%	34.3%	30.0%
*College graduate or above*	17.6%	23.3%	37.6%	45.6%
*Missing*	30.0%	26.1%	20.3%	17.1%

**Table 2:** Anthropometric characteristics of study population by gender.

	Male							
	White	Black	Asian	Hispanic	Other	ANOVA (p-value)	Group	Differences
	n=1283	n=1351	n=187	n=65	n=85			
Age (yrs)	56.99±11.11	52.38±6.58	57.56±10.50	52.26±7.82	51.37±6.96	<.0001	[W,A] > [B,H,O]	
Body fat (%)	26.36±5.47	23.85±6.20	25.56±4.06	26.25±5.19	23.33±5.53	<.0001	[W,H,A] > [B,O]	
BMI	27.70±5.02	27.07±5.54	24.69±2.99	28.53±4.29	25.49±4.59	<.0001	[H,W] > B > [O,A]	
WHR	0.93±0.07	0.89±0.07	0.89±0.05	0.92±0.06	0.91±0.07	<.0001	[W,H] > [B,A] W > O	
ASM/h^2^	8.84±1.27	9.36±1.46	8.11±0.98	9.15±1.22	8.66±1.22	<.0001	B > [H,W,O] > A	
	
	Female							
	White	Black	Asian	Hispanic	Other	ANOVA (p-value)	Group	Differences
	n=3018	n=1135	n=336	n=125	n=77			
	
Age (yrs)	58.70±10.55	54.26±8.49	55.13±9.27	55.14±9.73	53.60±8.39	<.0001	W > [H,A,B,O]	
Body fat (%)	37.73±6.42	39.72±6.26	36.24±4.74	37.32±5.59	38.59±6.07	<.0001	[B,O] > W > [H,A]	
BMI	27.22±6.36	31.69±7.48	24.24±4.04	27.33±4.87	29.02±6.72	<.0001	B>O,H,W>A O > W	
WHR	0.82±0.07	0.86±0.07	0.84±0.07	0.84±0.07	0.84±0.07	<.0001	[B,H],A > W B > A,[O,W]	
ASM/h^2^	6.93±1.19	8.21±1.53	6.36±0.91	6.99±0.88	7.43±1.38	<.0001	B>O>H>W>A	

**Table 3:** Prevalence rates of obesity, sarcopenia, and sarcopenic obesity by gender.

	Men						Women					
	
	Total n=2971	White n=1283	Black n=1351	Asian n=187	Hispanic n=65	Other n=85	Total n=4691	White n=3018	Black n=1135	Asian n=336	Hispanic n=125	Other n=77
BMI												
*Normal*	38.3%	32.7%	40.6%	58.8%	16.9%	55.3%	39.2%	44.4%	18.0%	66.7%	36.0%	31.2%
*Overweight*	36.8%	39.8%	33.8%	36.4%	47.7%	29.4%	28.7%	29.0%	27.4%	26.5%	37.6%	27.3%
*Obese*	25.0%	27.4%	25.5%	4.8%	35.4%	15.3%	32.2%	26.6%	54.6%	6.9%	26.4%	41.6%
WHR												
*Normal*	46.2%	33.9%	56.9%	52.9%	30.8%	56.5%	59.5%	65.6%	44.6%	56.9%	54.4%	61.0%
*Obese*	53.9%	66.1%	43.1%	47.1%	69.2%	43.5%	40.5%	34.4%	55.4%	43.2%	45.6%	39.0%
Sarcopenia												
*Normal*	82.4%	81.0%	87.1%	59.4%	89.2%	76.5%	86.3%	84.1%	96.4%	69.9%	90.4%	90.9%
*Sarcopenic*	17.6%	19.0%	13.0%	40.6%	10.8%	23.5%	13.7%	15.9%	3.6%	30.1%	9.6%	9.1%
Sarcopenic Obesity												
*Sarcopenic Obesity*	7.0%	9.3%	3.7%	14.4%	6.2%	9.4%	2.5%	2.5%	0.9%	8.0%	1.6%	3.9%
*Sarcopenia Only*	10.6%	9.7%	9.3%	26.2%	4.6%	14.1%	11.2%	13.4%	2.7%	22.0%	8.0%	5.2%
*Obesity Only*	46.9%	56.8%	39.4%	32.6%	63.1%	34.1%	38.0%	31.9%	54.5%	35.1%	44.0%	35.1%
*Normal*	35.6%	24.2%	47.7%	26.7%	26.2%	42.4%	48.4%	52.2%	41.9%	34.8%	46.4%	55.8%

**Table 4:** Associations of various social determinant factors with obesity, sarcopenia, and sarcopenic obesity.

	Male (n=2971)			Female (n=4691)		
	Sarcopenic Obesity	Sarcopenia Only	Obesity Only	Sarcopenic Obesity	Sarcopenia Only	Obesity Only
Age						
*40–49 years old*	-	-	-	-	-	-
*50–59 years old*	3.54***	1.27	1.67***	2.26*	1.42*	1.46***
*60–69 years old*	7.45***	2.88***	2.79***	2.86*	2.17***	1.82***
*70+ years old*	18.33***	2.93**	3.09***	4.96***	2.23***	1.72***
Ethnicity						
*White*	-	-	-	-	-	-
*Black*	0.21***	0.38***	0.46***	0.58	0.19***	1.60***
*Other*	0.85	1.60	0.49***	3.44***	1.83**	1.31
Salary						
*Less than $20,000*	2.78**	2.31*	1.07	1.65	1.49*	1.92***
*$20,000–$59,999*	1.29	1.03	1.22	1.24	0.85	1.26*
*$60,000 or more*	-	-	-	-	-	-
Education						
*< H.S. degree*	1.26	1.51	1.15	2.48	1.02	1.51**
*H.S. degree – some college*	1.52	1.34	1.30	2.68**	0.87	1.17
*College graduate or above*	-	-	-	-	-	-

Key:

* p < .05

** p < .01

*** p < .001

## References

[1] Prohaska TR, Anderson LA, Binstock RH. Public health for an aging society Baltimore, MD: Johns Hopkins University Press; 2012.

[2] Hetzel LS, Smith A. The 65 years and over population: 2000 http://www.census.gov/prod/2001pubs/c2kbr01-10.pdf US Census Bureau; 2001.

[3] Colby SL, Ortman JM. Projections of the size and composition of the U.S. population: 2014 to 2060 http://www.census.gov/content/dam/Census/library/publications/2015/demo/p25-1143.pdf US Census Bureau; 2015.

[4] Baumgartner RN, Koehler KM, Gallagher D, Romero L, Heymsfield SB, Ross RR, Garry PJ, Lindeman RD. Epidemiology of sarcopenia among the elderly in New Mexico. Am J Epidemiol 1999;147(8):755–63. 10.1093/oxfordjournals.aje.a009520 9554417

[5] Rosenberg IH. Sarcopenia: origins and clinical relevance. J Nutr 1997;127(5S):990S–1S. 9164280 10.1093/jn/127.5.990S

[6] Dutta C Significance of sarcopenia in the elderly. J Nutr 1997;127(5S):992S–3S. 9164281 10.1093/jn/127.5.992S

[7] Janssen I Evolution of sarcopenia research. Appl Physiol Nutr Metab 2010;35(5):707–12. 20962927 10.1139/H10-067

[8] Lammes E, Akner G. Repeated assessment of energy and nutrient intake in 52 nursing home residents. J Nutr Health Aging 2006;10(3):222–30. 16622584

[9] Janssen I, Heymsfield SB, Ross R. Low relative skeletal muscle mass (sarcopenia) in older persons is associated with functional impairment and physical disability. J Am Geriatr Soc 2002;50:889–96. 12028177 10.1046/j.1532-5415.2002.50216.x

[10] Prado CM, Wells JC, Smith SR, Stephan BC, Siervo M. Sarcopenic obesity: A Critical appraisal of the current evidence. Clin Nutr 2012;31(5):583–601. 22809635 10.1016/j.clnu.2012.06.010

[11] Zamboni M, Mazzali G, Fantin F, Rossi A, Di Francesco V. Sarcopenic obesity: a new category of obesity in the elderly. Nutr Metab Cardiovasc Dis 2008;18(5):388–95. 18395429 10.1016/j.numecd.2007.10.002

[12] Batsis JA, Barre LK, Mackenzie TA, Pratt SI, Lopez-Jimenez F, Bartels SJ. Variation in the prevalence of sarcopenia and sarcopenic obesity in older adults associated with different research definitions: dual-energy X-ray absorptiometry data from the National Health and Nutrition Examination Survey 1999–2004. J Am Geriatr Soc 2013;61(6):974–80. 23647372 10.1111/jgs.12260

[13] Dominguez LJ, Barbagallo M. The cardiometabolic syndrome and sarcopenic obesity in older persons. Journal of the cardiometabolic syndrome. J Cardiometab Syndr 2007;2(3):183–9. 17786082 10.1111/j.1559-4564.2007.06673.x

[14] Baumgartner RN. Body composition in healthy aging. Ann N Y Acad Sci 2000;904:437–48. 10865787 10.1111/j.1749-6632.2000.tb06498.x

[15] Chung JY, Kang HT, Lee DC, Lee HR, Lee YJ. Body composition and its association with cardiometabolic risk factors in the elderly: a focus on sarcopenic obesity. Arch Gerontol Geriatr 2013;56(1):270–8. 23079031 10.1016/j.archger.2012.09.007

[16] Han K, Park YM, Kwon HS, Ko SH, Lee SH, Yim HW, Lee WC, Park YG, Kim MK, Park YM. Sarcopenia as a determinant of blood pressure in older Koreans: findings from the Korea National Health and Nutrition Examination Surveys (KNHANES) 2008–2010. PLoS One 2014;9(1):e86902. 24489804 10.1371/journal.pone.0086902 PMC3906091

[17] Baek SJ, Nam GE, Han KD, Choi SW, Jung SW, Bok AR, Kim YH, Lee KS, Han BD, Kim DH. Sarcopenia and sarcopenic obesity and their association with dyslipidemia in Korean elderly men: the 2008–2010 Korea National Health and Nutrition Examination Survey. J Endocrinol Invest 2014;37(3):247–60. 24615361 10.1007/s40618-013-0011-3

[18] Kohara K Sarcopenic obesity in aging population: Current status and future directions for research. Endocrine 2014;45(1):15–25. 23821364 10.1007/s12020-013-9992-0

[19] Muscaritoli M, Anker SD, Argilés J, Aversa Z, Bauer JM, Biolo G, Boirie Y, Bosaeus I, Cederholm T, Costelli P, Fearon KC, Laviano A, Maggio M, Rossi Ranelli F, Schneider SM, Schols A, Sieber CC. Consensus definition of sarcopenia, cachexia and pre-cachexia: joint document elaborated by Special Interest Groups (SIG) “cachexia-anorexia in chronic wasting diseases” and “nutrition in geriatrics”. Clin Nutr 2010;29(2):154–9. 20060626 10.1016/j.clnu.2009.12.004

[20] Cruz-Jentoft AJ, Baeyens JP, Bauer JM, Boirie Y, Cederholm T, Landi F, Martin FC, Michel JP, Rolland Y, Schneider SM, Topinková E, Vandewoude M, Zamboni M, European Working Group on Sarcopenia in Older People. Sarcopenia: European consensus on definition and diagnosis: Report of the European Working Group on Sarcopenia in Older People. Age Ageing 2010;39(4):412–23. 20392703 10.1093/ageing/afq034 PMC2886201

[21] Fielding RA, Vellas B, Evans WJ, Bhasin S, Morley JE, Newman AB, Abellan van Kan G, Andrieu S, Bauer J, Breuille D, Cederholm T, Chandler J, De Meynard C, Donini L, Harris T, Kannt A, Keime Guibert F, Onder G, Papanicolaou D, Rolland Y, Rooks D, Sieber C, Souhami E, Verlaan S, Zamboni M. Sarcopenia: An undiagnosed condition in older adults. Current consensus definition: prevalence, etiology, and consequences. International working group on sarcopenia. J Am Med Dir Assoc 2011;12(4):249–56. 21527165 10.1016/j.jamda.2011.01.003 PMC3377163

[22] Chen LK, Liu LK, Woo J, Assantachai P, Auyeung TW, Bahyah KS, Chou MY, Chen LY, Hsu PS, Krairit O, Lee JS, Lee WJ, Lee Y, Liang CK, Limpawattana P, Lin CS, Peng LN, Satake S, Suzuki T, Won CW, Wu CH, Wu SN, Zhang T, Zeng P, Akishita M, Arai H. Sarcopenia in Asia: Consensus report of the Asian Working Group for Sarcopenia. J Am Med Dir Assoc 2014;15(2):95–101. 24461239 10.1016/j.jamda.2013.11.025

[23] Studenski SA, Peters KW, Alley DE, Cawthon PM, McLean RR, Harris TB, Ferrucci L, Guralnik JM, Fragala MS, Kenny AM, Kiel DP, Kritchevsky SB, Shardell MD, Dam TT, Vassileva MT. The FNIH sarcopenia project: Rationale, study description, conference recommendations, and final estimates. J Gerontol A Biol Sci Med Sci 2014;69(5):547–58. 24737557 10.1093/gerona/glu010 PMC3991146

[24] Morley JE, Abbatecola AM, Argiles JM, Baracos V, Bauer J, Bhasin S, Cederholm T, Coats AJ, Cummings SR, Evans WJ, Fearon K, Ferrucci L, Fielding RA, Guralnik JM, Harris TB, Inui A, Kalantar-Zadeh K, Kirwan BA, Mantovani G, Muscaritoli M, Newman AB, Rossi-Fanelli F, Rosano GM, Roubenoff R, Schambelan M, Sokol GH, Storer TW, Vellas B, von Haehling S, Yeh SS, Anker SD, Society on Sarcopenia, Cachexia and Wasting Disorders Trialist Workshop. Sarcopenia with limited mobility: An international consensus. J Am Med Dir Assoc 2011;12(6):403–9. 21640657 10.1016/j.jamda.2011.04.014 PMC5100674

[25] Prentice AM, Jebb SA. Beyond body mass index. Obes Rev 2001;2(3):141–7. 12120099 10.1046/j.1467-789x.2001.00031.x

[26] Dulloo AG, Jacquet J, Solinas G, Montani JP, Schutz Y. Body composition phenotypes in pathways to obesity and the metabolic syndrome. Int J Obes (Lond) 2010;34(S2):S4–17. 21151146 10.1038/ijo.2010.234

[27] Norgan NG. Laboratory and field measurements of body composition. Public Health Nutr 2005;8(7A):1108–22. 16277823 10.1079/phn2005799

[28] Jih J, Mukherjea A, Vittinghoff E, Nguyen TT, Tsoh JY, Fukuoka Y, Bender MS, Tseng W, Kanaya AM. Using appropriate body mass index cut points for overweight and obesity among Asian Americans. Prev Med 2014;65:1–6. 24736092 10.1016/j.ypmed.2014.04.010 PMC4217157

[29] WHO Expert Consultation. Appropriate body-mass index for Asian populations and its implications for policy and intervention strategies. Lancet 2004;363(9403):157–63. 14726171 10.1016/S0140-6736(03)15268-3

[30] Hurst L, Stafford M, Cooper R, Hardy R, Richards M, Kuh D. Lifetime socioeconomic inequalities in physical and cognitive aging. Am J Public Health 2013;103(9):1641–8. 23865666 10.2105/AJPH.2013.301240 PMC3780680

[31] Hwang B, Lim JY, Lee J, Choi NK, Ahn YO, Park BJ. Prevalence rate and associated factors of sarcopenic obesity in korean elderly population. J Korean Med Sci 2012;27(7):748–55. 22787369 10.3346/jkms.2012.27.7.748 PMC3390722

[32] Fanelli Kuczmarski M, Mason MA, Beydoun MA, Allegro D, Zonderman AB, Evans MK. Dietary patterns and sarcopenia in an urban African American and White population in the United States. J Nutr Gerontol Geriatr 2013;32(4):291–316. 24224938 10.1080/21551197.2013.840255 PMC4669047

[33] Alexandre TdaS, DuarteYA, SantosJL, WongR, LebraoML. Prevalence and associated factors of sarcopenia among elderly in Brazil: findings from the SABE study. J Nutr Health Aging 2014;18(3):284–90.2462675610.1007/s12603-013-0413-0

[34] He H, Liu Y, Tian Q, Papasian CJ, Hu T, Deng HW. Relationship of sarcopenia and body composition with osteoporosis. Osteoporos Int 2016;27(2):473–82. 26243357 10.1007/s00198-015-3241-8

[35] Cauley JA. An Overview of Sarcopenic Obesity. J Clin Densitom 2015;18(4):499–505. 26141163 10.1016/j.jocd.2015.04.013

[36] Division of Nutrition, Physical Activity, and Obesity, National Center for Chronic Disease Prevention and Health Promotion. About adult BMI Centers for Disease Control and Prevention 2015 http://www.cdc.gov/healthyweight/assessing/bmi/adult_bmi/index.html.

[37] World Health Organization. Waist circumference and waist-hip ratio: Report of a WHO expert consultation, Geneva, 8–11 December 2008 Geneva: WHO Document Production Services 2011 http://apps.who.int/iris/bitstream/10665/44583/1/9789241501491_eng.pdf.

[38] Wu IC, Lin CC, Hsiung CA, Wang CY, Wu CH, Chan DC, Li TC, Lin WY, Huang KC, Chen CY, Hsu CC, Sarcopenia and Translational Aging Research in Taiwan Team. Epidemiology of sarcopenia among community-dwelling older adults in Taiwan: A pooled analysis for a broader adoption of sarcopenia assessments. Geriatr Gerontol Int 2014;14(S1):52–60. 24450561 10.1111/ggi.12193

[39] Lim S, Kim JH, Yoon JW, Kang SM, Choi SH, Park YJ, Kim KW, Lim JY, Park KS, Jang HC. Sarcopenic obesity: Prevalence and association with metabolic syndrome in the Korean Longitudinal Study on Health and Aging (KLoSHA). Diabetes Care 2010;33(7):1652–4. 20460442 10.2337/dc10-0107 PMC2890376

[40] ParkS, HamJO, LeeBK. A positive association between stroke risk and sarcopenia in men aged >/= 50 years, but not women: Results from the Korean National Health and Nutrition Examination Survey 2008–2010. J Nutr Health Aging 2014;18(9):806–12.2538995810.1007/s12603-014-0553-x

[41] Sanada K, Miyachi M, Tanimoto M, Yamamoto K, Murakami H, Okumura S, Gando Y, Suzuki K, Tabata I, Higuchi M. A cross-sectional study of sarcopenia in Japanese men and women: Reference values and association with cardiovascular risk factors. Eur J Appl Physiol 2010;110(1):57–65. 20390291 10.1007/s00421-010-1473-z

